# Prevalence of Workplace Bullying in the Syrian Graduate Medical Education System During COVID-19 Pandemic and Civil War: A National Cross-Sectional Study

**DOI:** 10.1192/bjo.2022.220

**Published:** 2022-06-20

**Authors:** Mhd Obai Alchallah, Homam Alolabi, Fatema Mohsen, Nawras AlHalabi, Ghadir Abbas, Youssef Latifeh, Bisher Sawaf

**Affiliations:** 1 Faculty of Medicine, Syrian Private University, Damascus, Syrian Arab Republic; 2 Department of Ophthalmology, Al- Mouwassat University Hospital, Damascus University, Damascus, Syrian Arab Republic; 3 Department of Psychiatry, Al- Mouwassat University Hospital, Damascus University, Damascus, Syrian Arab Republic; 4 Department of Psychiatry, Faculty of Medicine, Syrian Private University, Damascus, Syrian Arab Republic; 5 Internal Medicine Department, Hamad General Hospital, Hamad Medical Corporation, Doha, Qatar

## Abstract

**Aims:**

Workplace Bullying (WPB) is a severe stressor that can negatively impact an individual's physical and psychological health. WPB, a type of occupational violence, is the third leading cause of death in the workplace worldwide. This study delivers an estimated prevalence of bullying among healthcare practitioners in the Syrian graduate medical education system and to explore its prevalence within socio-demographic subgroups.

**Methods:**

A cross-sectional study was conducted in Damascus, during the Syrian war crisis. A total of 478 residents and fellows fully completed the survey. Respondents completed questions regarding socio-demographic information and workplace bullying.

**Results:**

Of 478 respondents, 267 (55.9%) were males. The majority (89%) reported being subjected to workplace bullying, and (92%) of them witnessed their colleagues being bullied. Supervisor/attendings (45%), and peer/resident (40%) were the most frequent source of perceived bullying followed by supervisor/consultant (34.5%), and Patients (33.5%). Attempts to belittle and undermine work 434 (90.7%) was the most frequently reported bullying behavior. Specific bullying behaviors were more reported by males, ＜170 cm height, ≥ 25 BMI kg/m2, and postgraduate year 1 (PGY) participants. Credible published national data regarding the number of Syrian medical residents are not available to evaluate the representativeness of our sample.

**Conclusion:**

Many participants reported experiencing bullying in the Syrian graduate medical education programs. Enforcing anti-bullying policies, closely monitoring work environments, and encouraging anonymous reporting of workplace bullying, is crucial to eliminate these behaviors in the healthcare system. A longitudinal study should be conducted to gain more knowledge and insight into workplace bullying among healthcare practitioners.

